# Perceptions of friendship, peers and influence on adolescent smoking according to tobacco control context: a systematic review and meta-ethnography of qualitative research

**DOI:** 10.1186/s12889-022-14727-z

**Published:** 2023-03-03

**Authors:** H. J. Littlecott, G. F. Moore, R. E. Evans, G. J. Melendez-Torres, M. McCann, H. Reed, M. Mann, F. Dobbie, S. Jennings, C. Donaldson, J. Hawkins

**Affiliations:** 1 Pettenkofer School of Public Health (PSPH), Institute for Medical Information Processing, Biometry and Epidemiology (IBE), LMU Munich, Elisabeth-Winterhalter-Weg 6, Munich, 81377 Germany; 2 Centre for Development, Evaluation, Complexity and Implementation in Public Health Improvement (DECIPHer), School of Social Sciences, Cardiff University, 1-3 Museum Place, Cardiff, CF10 3BD Wales, UK; 3 Peninsula Technology Assessment Group (PenTAG), South Cloisters, University of Exeter, St Luke’s Campus, Heavitree Road, Exeter, EX1 2LU UK; 4 MRC/CSO Social and Public Health Sciences Unit, University of Glasgow, Berkeley Square, 99 Berkeley Street, Glasgow, G3 7HR UK; 5 Specialist Unit for Review Evidence, Cardiff University, 6th Floor, Neuadd Meirionnydd, Heath Park Campus, Cardiff, CF14 4YS UK; 6 Usher Institute- University of Edinburgh, Doorway 1, Old Medical School, Teviot Place, Edinburgh, EH8 9AG UK; 7 Teaching and Learning for Health Professionals, Bristol Medical School, Faculty of Health Sciences, University of Bristol, 39-41 St Michael’s Hill, Bristol, BS2 8EZ UK

**Keywords:** Smoking, Tobacco control, Adolescents, Schools, Friendship, Peer influence, Systematic review, meta-ethnography

## Abstract

**Background:**

A relationship between smoking and interpersonal influences has been well established within the literature. There have been cultural shifts in denormalisation and a reduction in tobacco smoking in many countries. Hence there is a need to understand social influences on adolescents’ smoking across smoking normalisation contexts.

**Methods:**

The search was conducted in July 2019 and updated in March 2022 within 11 databases and secondary sources. Search terms included schools, adolescents, smoking, peers, social norms and qualitative research. Screening was conducted by two researchers independently and in duplicate. Study quality was assessed using the eight-item Evidence for Policy and Practice Information and Co-ordinating Centre (EPPI-centre) tool for the appraisal of qualitative studies. Results were synthesised using a meta-narrative lens for meta-ethnography and compared across smoking normalisation contexts.

**Results:**

Forty one studies were included and five themes were developed, mapping onto the socio ecological model. The social processes by which adolescents take up smoking differed according to a mixture of school type, peer group structure and the smoking culture within the school, as well as the wider cultural context. Data available from smoking denormalised contexts, described changes in social interactions around smoking to cope with its stigmatisation. This was manifested through i) direct peer influence, whereby subtle techniques were employed, ii) group belonging whereby smoking was less likely to be seen as a key determinant of group membership and smoking was less commonly reported to be used as a social tool, and iii) popularity and identity construction, whereby smoking was perceived more negatively in a denormalised context, compared with a normalised context.

**Conclusions:**

This meta-ethnography is the first study to demonstrate, drawing on international data, that peer processes in adolescent smoking may undergo changes as smoking norms within society change. Future research should focus on understanding differences across socioeconomic contexts, to inform the adaptation of interventions.

**Supplementary Information:**

The online version contains supplementary material available at 10.1186/s12889-022-14727-z.

## Introduction

The relationship between smoking and peers has been well established within the literature, with a review of qualitative research having identified interpersonal influences on smoking, including a desire for peer acceptance and a sense of belonging [1]. Previous research has also established that smoking attitudes and behaviours of adolescents and their peers may be influenced at multiple socioecological levels, which interact with interpersonal influences to affect behaviour. For example, adolescent smoking has been found to be associated with intrapersonal characteristics such as individual level socioeconomic status [2], self-esteem [3] and the construction of ‘cool’ and ‘popular’ identities [4]. At organisational and community levels, influences on smoking might include school level socioeconomic status, the development of subculture identities within schools [5, 6] and closeness of the school community [7] whereby smoking uptake may diffuse through close knit peer communities easily. However, most existing evidence has been captured prior to the introduction of comprehensive smoking bans, in contexts where tobacco smoking remains highly normalised [8, 9]. Despite a large decrease in smoking prevalence, socioeconomic inequality has prevailed [10–12]. For example, young people living in the 20% most deprived areas in England were found to be up to three times more likely to be smokers than their counterparts in the 20% least deprived areas [13]. The evidence above demonstrates the importance of addressing structural determinants and considering tobacco control context when intervening to reduce or prevent smoking.

The epidemiological context of adolescent tobacco smoking has changed, with prevalence of youth smoking decreasing to its lowest level since the all-time highs at the turn of the 21st century [14]. Various legislation linked to pricing and tax, advertising, packaging and labelling, and the banning of smoking in public places have been variably implemented in different countries [15] perhaps in part caused by and causing a cultural shift towards smoking denormalisation. Such denormalisation may have led to the reduction in effectiveness of anti-smoking policies in UK schools. As fewer students already smoke, students exist in spaces where tobacco norms have changed and those who continue to smoke may be less influenced by the school norms [16]. Despite this, many key interventions to target adolescent smoking that have been found to be effective, are still based on harnessing peer influence and changing pro-smoking norms within the school context [7]. Therefore, it is vital for research to revisit understandings of whether, and how, peer influence and selection still functions to diffuse smoking attitudes and behaviours in school networks where smoking may be denormalised, and how stakeholder perceptions can contribute to a greater insight.

The influence of community context has been shown in intervention research where schools located in stable areas with high levels of community attachment had high smoking rates to begin with. It is assumed the closeness of students meant increased contact between peer educators and other students which led to increased intervention effects in these communities [7]. This assumption alludes to the influence of the student community on the relationship between smoking and peers and sets up a hypothesis that smoking uptake diffuses through close knit peer communities more easily. Thus, this has implications for the design of interventions to tackle smoking in different school contexts. Much of the research supporting the effectiveness of such interventions was conducted prior to the introduction of comprehensive tobacco legislation within these countries. Thus, there is a need to explore these claims with school stakeholders at different stages of the tobacco epidemic, with different levels of tobacco normalisation.

### Objectives

The need to understand health inequalities in relation to adolescents’ smoking attitudes suggests that a systematic review of qualitative research could contribute meaningfully. Changes in the legislative context, can be used as a proxy for the extent or context of tobacco denormalization within each country. In particular, a meta-ethnography, whereby variation in tobacco denormalisation contexts are taken into account could help to elicit overarching theoretical interpretations and understanding of the included primary studies, that are bigger than the sum of their parts [17]. This systematic review and meta-ethnography builds upon previous research by adding a focus on smoking normalisation contexts to address the following research question and sub-questions:

1. How do school students (age 11–18), school staff, parents, or other education professionals view peer influence on adolescent smoking attitudes and behaviours?

- How do these views vary over time according to the proximity of the introduction of comprehensive smoking legislation at the time of data collection?
- How do these views vary by individual and school-level socioeconomic status?

## Methods

### Protocol and registration

The systematic review protocol was registered with PROSPERO (CRD42019137358) in April 2020 where further details may be found [18]. The review is reported in accordance with the Preferred Reporting Items for Systematic Reviews and Meta Analyses (PRISMA) guidelines [19, 20] and the eMERGe meta-ethnography reporting guidance [21].

### Eligibility criteria

The search criteria were guided by the Sample, Phenomenon of Interest, Design, Evaluation, Research type (SPIDER) framework [22]. Publications meeting the criteria outlined in Table 1 were included.

**Table 1:** Eligibility criteria according to the SPIDER framework

SPIDER Framework	Description
Sample	• Studies that sought school students (age 11–18), school staff, parents or other education professionals’ views and were focused on whole population, or students of a low socioeconomic status. • Studies that focused on special populations, for example, cannabis smokers were excluded.
Phenomenon of Interest	• Studies that focused on friendship, peers, influence and selection. • Studies were excluded if they focused exclusively on waterpipe tobacco, e-cigarettes and other forms of nicotine inhalation as well as passive smoking and cessation studies.
Design	• Qualitative or mixed methods studies with a qualitative element including interviews, focus groups, and observations.
Evaluation	• Studies that sought participants’ views, perceptions or attitudes.
Research Type	• Date: Papers published using data collected during or after 1997. This is the year that adolescent smoking peaked in the US (30). Corresponding authors were contacted directly to request this information, where this was omitted in papers. • Language: No language or geographical limits were set, but comparisons were made within the analyses according to whether the data were collected before or after the introduction of comprehensive smoking legislation covering bans on smoking in all work places and public places, including restaurants and bars, in each respective country.

### Information sources and searches

Searches for abstracts, full-texts and conference proceedings were conducted on 12th July 2019 and updated on 4th March 2022 by the lead author (HL). The following bibliographic databases and a variety of secondary sources, including the reference lists of key included publications, were searched; CINAHL Plus with full text, Embase, MEDLINE, Education Resources Information Center (ERIC), British Education Index (BEI), Open Dissertations, Psycinfo, Scopus, Applied Social Science Index & Abstracts (ASSIA), Sociological Abstracts, and E-Theses Online Service (EThOS). The search was developed and refined in MEDLINE (Additional file 1) before adapting to the specifications of each database.

### Study selection

Identified studies were de-duplicated in Endnote and subsequently imported into Rayyan screening software. Each title and abstract was screened independently and in duplicate, followed by full text screening of a smaller subset of records, shared between three researchers (HL, HR, SJ). Discrepancies were resolved by a third reviewer (GJMT).

### Data extraction

A review data extraction form was developed and piloted with a subset of two studies. Full text extraction was conducted by two independent reviewers (HL, CD), who extracted the following data; title, year of publication, year of data collection, participant number and characteristics, setting and tobacco control context, study design and methods, analysis, results and conclusions.

### Quality assessment

All included studies were independently appraised for quality in duplicate, with workload shared between three researchers (HL, CD, GJMT). Study quality was assessed using the eight-item Evidence for Policy and Practice Information and Co-ordinating Centre (EPPI-centre) tool for the appraisal of qualitative studies [23], which includes domains focused on the rigour of sampling, data collection, and data analysis procedures. Further domains focused on whether findings were supported by the data and their level of breadth and depth, privilege of children’s perspectives, reliability/trustworthiness and usefulness. Studies were rated low, medium, or high according to the weight assigned for the trustworthiness of findings of each study for use in this review. Discrepancies were resolved by a third reviewer (GJMT). Further details are included in the review protocol [18].

### Synthesis

A meta-narrative lens was applied throughout the seven stages of meta-ethnographic synthesis. This novel approach was employed to obtain an understanding of how different paradigms may have influenced this field. Meta-narrative reviews focus on an unfolding storyline of how fields have changed over time, thus providing a methodology through which to understand true changes in the social influence of smoking over time. These changes are in line with legislation restricting smoking, and the extent to which methodological advances and paradigm shifts may have had a role in these advances in understanding and changing results [17]. This meta-narrative approach required that the location of studies according to their position on a narrative story line starting from contexts where smoking was highly normalised where comprehensive tobacco legislation was yet to be introduced, contexts that were nearing introduction, and extending to highly denormalised smoking contexts where comprehensive tobacco legislation had already been introduced.

Findings were synthesised by the lead author (HL), and were verified by others during the write up period. Studies were divided into eight groups (see Additional file 2 for table) according to the timing of data collection in relation to the introduction of comprehensive tobacco legislation in each respective country (10 + years before/no smoking ban introduced; 5–9 years before; 0–4 years before; or after the introduction of comprehensive tobacco legislation), combined with the quality rating (high quality or medium/low quality). Organisation by chronological groups, stratified by quality ensured that findings were not driven by low quality studies. The seven phases of meta-ethnography were undertaken; getting started, deciding what is relevant to the initial interest, reading the studies, determining how the studies are related, translating the studies into one another, synthesising translations and expressing the synthesis [21]. During phase seven, expressing the synthesis, findings within each group were organised using the socio-ecological model [24]. Within each level of this model, a lines of argument approach was employed to understand how the combination of individual findings contributed to a greater understanding than each individual study [21].

## Results

### Study selection

**Fig. 1:**
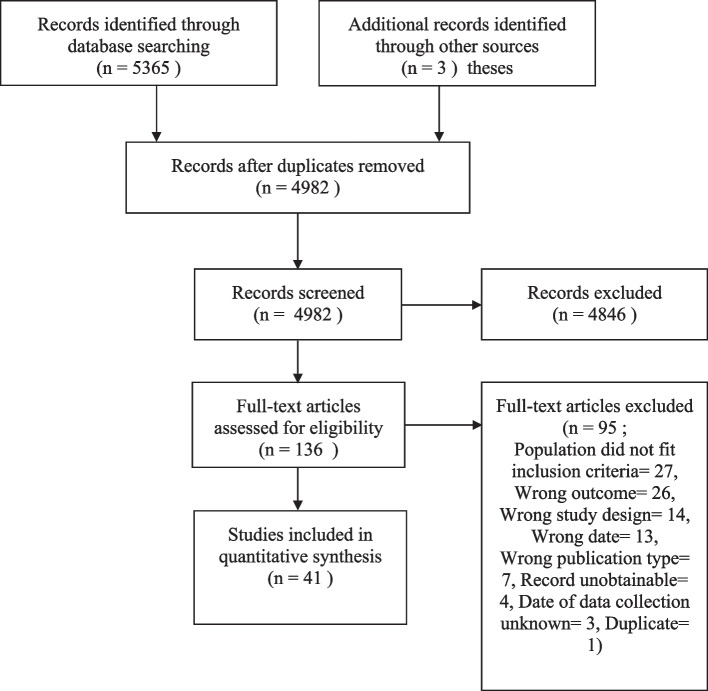
PRISMA flow diagram

The searches identified 5365 records (see PRIMSA Fig. 1). Forty one studies were included in the systematic review. As the date of data collection was required for the chronological analysis within this review, the authors of fourteen studies which did not specify the year of data collection were contacted for each of these studies, with ten responding to provide the year of data collection. Three did not respond and were therefore excluded from the review, one did not respond, but was still included due to there being no comprehensive smoking legislation introduced in the country and, therefore, being placed into the ‘before’ category.

### Overview of included studies

An overview of the characteristics of included studies and their methods and context are included in Tables 2 and 3.

**Table 2:** Characteristics of included studies

Author and year	Year of data collection	Participant characteristics			Country	Quality assessment
		**Age**	**Number**	**Socioeconomic status**		
Amos et al. (2007) [25]	2002	Range 15–16 years	46 ( 24 females and 22 males)	4 focus groups from middle class (ABC1) and 4 from working class (C2DE)	UK (Scotland)	HIGH
Arora et al. (2010) [26]	2005	Range 10–19 years	37 (6 females and 31 males)	2 low SES communities	India	MEDIUM
Baheiraei et al. (2018) [27]	2012	Range 15–18 years	11 females	Not recorded	Iran	MEDIUM
Baillie et al. (2005) [28]	2000-1	Range 14–18 years, mean 16 years	35 (17 females and 18 males)	Not recorded	Canada (British Columbia)	HIGH
Craciun et al. (2008) [29]	2005-6	Range14-15 years	30 (15 females and 15 males)	Not recorded	Romania	LOW
Denscombe et al. (2001) [30]	1997-8	Range 15–16 years	123 Focus groups, 20 interviews	Not recorded	UK (England)	HIGH
Denscombe et al. (2001b) [31]	1997-8	Range 15–16 years	123 Focus groups, 20 interviews	Not recorded	UK (England)	HIGH
Dijk et al. (2007) [32]	2003	Range15-17 years	101	Not recorded	Netherlands	MEDIUM
El Kazdouh et al. (2018) [33]	2016	Range 14–16 years	100	2 schools - one classed as “advantaged”, the other as “disadvantaged”	Morocco	HIGH
Fithria (2021) [34]	2019	Range 12–18 years	24 male students	Schools located in regions with a poverty level of 15.41%	Indonesia	LOW
Fraga et al. (2011) [35]	2003-4	Mean/range 13 years	30 (15 females and 15 males)	Not recorded	Portugal	LOW
Haines et al. (2009) [36]	2005-6	Range 16–19 years	25	No data collected but researchers say that most appeared to be mid to high SES	Canada (Toronto)	MEDIUM
Hong et al. (2015) [37]	2013	Range 13–18 years	12	Not recorded	Taiwan	MEDIUM
Ioannou et al. (2010) [38]	2002	Range 15–17 years	25 (13 females and 12 males)	States ‘diverse socioeconomic backgrounds’	Cyprus	MEDIUM
Jafari (2022) [39]	2020	Mean 16 years	20 females	Not recorded	Iran	MEDIUM
Johnson et al. (2003) [40]	2000-1	1st phase mean = 16, range 14–18; 2nd and 3rd phases mean = 16, range 13–19 years	1st phase 47 (29 females and 18 males); 2nd and 3rd phases − 25 (14 females and 11 males).	Not recorded	Canada (Vancouver)	HIGH
Lewis et al. (2013) [41]	2009	Range 11–18 years	52 (30 females and 22 males)	‘Disadvantaged community’ - “The youth club featured is situated in a former coal-mining village which, according to the index of multiple deprivation score (North East Public Health Observatory 2007), is amongst the 10 per cent most deprived wards in a county that is one of the most deprived in England. Unemployment levels are in the highest quintile (Durham County Council 2012) for the county.”	UK (England)	HIGH
Milton et al. (2008) [42]	2001	Range 9–11 years	76	Over half of the cohort lived in low-income families, and 82% lived in the most deprived quartile (the poorest quarter of addresses) in the northwest of England as calculated using Townsend’s indices of deprivation.	UK (England)	HIGH
Mishra et al. (2005) [43]	2002	Range 10–16 years	435 (181 females and 254 males)	Government run schools with low-medium SES; private schools with medium-high SES were included.	India	HIGH
Mitschke et al. (2008) [44]	2006	Range 10–14 years	54 (35 females and 19 males)	Not recorded	USA (Hawaii)	MEDIUM
Mutaz (2020) [45]	unknown	Range 12–16 years	103 males	Not recorded	Saudi Arabia	MEDIUM
Niknami et al. (2008) [46]	2004-5	Range 10–47 years	62 (92% male)	Not recorded	Iran	HIGH
Nwafor et al. (2012) [47]	2008	Not recorded	40 male	Not recorded	Nigeria	LOW
Perez-Milena et al. (2011) [48]	2008-9	Range 12–18 years	44 (6 focus groups ranging from 17-78% female)	Within the six focus groups, there were between 0–33% composition of the lowest socioeconomic group, between 42–83% middle and 11–50% highest.	Spain	MEDIUM
Peterson et al. (2019) [49]	2012-13	Range 12–16 years	81	Students are rated high/medium/low SES but no info on how this has been done.	Uruguay	MEDIUM
Plano Clark et al. (2002) [50]	1999	Mean 16 years	205 (plus 66 student co-researchers)	Not recorded	USA (Newbraska)	HIGH
Plumridge et al. (2002) [51]	1999	Range 14–15 years	42	School of relatively high socio-economic catchment (decile 8 ranking)	New Zealand	MEDIUM
Povlsen et al. (2018) [52]	2013	Range 13–16 years	71 (36 females and 35 males)	2 public and 2 private schools/castes recorded	Nepal	MEDIUM
Rothwell et al. (2011) [53]	2007	Mean 17 years, range 14–17 years	28	Not recorded	USA (Utah)	MEDIUM
Sanchez Martinez et al. (2008) [54]	2005	Range 16–17 years	14	Not recorded	Mexico	LOW
Schreuders et al. (2019) [55]	2016-17	Ranges: focus groups: 14–17 years old; interviews 15–18 years old	22 for focus groups; 14 for interviews	1 vocational school and one mid-level theoretical school	Netherlands	HIGH
Stewart-Knox et al. (2005) [56]	1997–2000	Ranges: Year 1: 11–12 years old; year 2: 12–13 years old; year 3: 13–14 + years	Year 1: 102 (52 females;50 males); Year 2: 51 (28 females;23 males); Year 3: 39 (22 females; 17 males)	Not recorded	UK (Northern Ireland)	HIGH
Stjerna et al. (2004) [57]	1999	Range 14–15 years	43 (25 females and 18 males)	Schools had ‘average SES structure’	Sweden	MEDIUM
Talip et al. (2016) [58]	2015	Mean 14 years, range 13–17 years	43 males	Not recorded	Brunei	MEDIUM
Tamvakas et al. (2010) [59]	2009	Mean 15 years, range 14–16 years	31 (14 females and 17 males)	Not recorded	Greece	MEDIUM
Tohid et al. (2011) [60]	2008-10	Mean/range 16 years	26 (3 females and 23 males)	Not recorded	Malaysia	MEDIUM
Treacy et al. (2007) [61]	1997	Longitudinal - yearly from 11–12 to 15–16 years	1st round 78(44 females and 34 males); 2nd round 48; 3rd round 19; 4th rounds 33	Most of sample from working-class areas of Dublin	Republic of Ireland	HIGH
Turner et al. (2006) [62]	2000-1	Mean/range 13 years	136	Both schools served disadvantaged populations	UK (Scotland)	HIGH
Vasquez et al. (2018) [63]	2015	Range 9–19 years	49 (60% males)	90% eligible for free school lunch	USA (Texas)	HIGH
Woodgate et al. (2015) [64]	2007-10	Mean 14.5 years, range 11–19 years	75	72% identified as middle class	Canada (Western Canadian Province)	HIGH
Yuksel et al. (2005) [65]	2001-2	Median 16 years	52 youth (19 females and 33 males) + 24 adults (teachers/school counsellors/parents)	Not reported	Turkey	MEDIUM

**Table 3:** Study methods and smoking legislative context

Author and year	Data collection methods	Analysis	Substance focus	Country and year of smoking ban	Synthesis category
Amos et al. (2007) [25]	Face to face single sex focus groups	Thematic	Smoking only	Scotland (UK) 2006	0–4 years before
Arora et al. (2010) [26]	Face to face focus groups	Thematic	Smoking and smoke-free tobacco	India no comprehensive ban	10 + years before
Baheiraei et al. (2018) [27]	Telephone semi-structured interviews	Thematic (constant comparative analysis/content analysis)	Smoking only	Iran 2007	After
Baillie et al. (2005) [28]	Face to face semi-structured interviews	Thematic (narrative enquiry)	Smoking only	Canada (British Columbia) 2008	5–9 years before
Craciun et al. (2008) [29]	Face to face semi-structured interviews	Thematic (content analysis)	Smoking only	Romania 2016	10 + years before
Denscombe et al. (2001) [30]	Face to face focus groups and semi-structured interviews	Thematic	Smoking only	England (UK) 2007	5–9 years before
Denscombe et al. (2001b) [31]	Face to face focus groups and semi-structured interviews	Thematic	Smoking only	England (UK) 2007	5–9 years before
Dijk et al. (2007) [32]	Face to face group interviews	Thematic	Smoking only	Netherlands 2008	5–9 years before
El Kazdouh et al. (2018) [33]	Face to face single sex focus groups	Thematic (inductive)	Substance use	Morocco no comprehensive ban	10 + years before
Fithria (2021) [34]	Face to face focus groups	Thematic (inductive content analysis)	Smoking only	Indonesia no comprehensive ban	10 + years before
Fraga et al. (2011) [35]	Face to face semi-structured interviews	Thematic (content analysis)	Smoking only	Portugal no comprehensive ban	0–4 years before
Haines et al. (2009) [36]	Face to face semi-structured interviews	Thematic	Smoking and other substances	Canada (Toronto) 2015	0–4 years before
Hong et al. (2015) [37]	Face to face semi-structured interviews and focus groups	Thematic (Colaixxi’s method)	Smoking only	Taiwan 2009	After
Ioannou et al. (2010) [38]	Face to face unstructured interviews	Thematic (content analysis/grounded theory)	Smoking only	Cyprus 2010	5–9 years before
Jafari (2022) [39]	Face to face semi-structured interviews	Thematic (content analysis)	Smoking only	Iran 2007	After
Johnson et al. (2003) [40]	Face to face semi-structured interviews (secondary analysis and primary data collection) + free pile and sort	Thematic	Smoking only	Canada (Vancouver) 2015	10 + years before
Lewis et al. (2013) [41]	Ethnography	Thematic (open coding approach)	Smoking only	UK (England) 2007	After
Milton et al. (2008) [42]	Face to face focus groups and semi-structured interviews	Thematic	Smoking only	UK (England) 2007	5–9 years before
Mishra et al. (2005) [43]	Face to face focus groups	Thematic	Tobacco in various forms	India, no comprehensive ban	10 + years before
Mitschke et al. (2008) [44]	Face to face focus groups	Thematic	Smoking only	Honolulu, Hawaii, USA 2006	0–4 years before
Mutaz (2020) [45]	Face to face focus groups	Thematic	Smoking only	Saudi Arabia, no comprehensive ban	10 + years before
Niknami et al. (2008) [46]	Face to face semi-structured interviews, focus groups and written narratives	Thematic (content analysis)	Smoking only	Iran 2007	0–4 years before
Nwafor et al. (2012) [47]	Face to face focus groups	Not stated clearly	Smoking only	Nigeria no comprehensive ban	10 + years before
Perez-Milena et al. (2011) [48]	Face to face focus groups	Content analysis	Smoking only	Spain 2011	0–4 years before
Peterson et al. (2019) [49]	Face to face focus groups	Thematic (constant comparison)	Smoking only	Uruguay 2006	After
Plano Clark et al. (2002) [50]	Face to face focus groups	Thematic	Primarily smoking, but also included smokeless tobacco	USA (Newbraska) 2009	5–9 years before
Plumridge et al. (2002) [51]	Face to face focus groups	Thematic	Smoking only	New Zealand 2004	5–9 years before
Povlsen et al. (2018) [52]	Face to face single sex focus groups	Thematic (content analysis)	Smoking only	Nepal 2011	After
Rothwell et al. (2011) [53]	Face to face focus groups	Thematic	Smoking and chewing tobacco	USA (Utah) 2007	0–4 years before
Sanchez Martinez et al. (2008) [54]	Face to face semi-structured interviews	Thematic (content analysis)	Smoking only	Mexico 2008	10 + years before
Schreuders et al. (2019) [55]	Face to face focus groups and semi-structured interviews	Thematic (framework analysis)	Smoking only	The Netherlands 2008	After
Stewart-Knox et al. (2005) [56]	Face to face semi-structured interviews	Thematic (content analysis/grounded theory)	Smoking only	UK (Northern Ireland) 2007	5–9 years before
Stjerna et al. (2004) [57]	Face to face single sex focus groups	Thematic (inductive/discursive analysis)	Tobacco, including snuff	Sweden 2005	10 + years before
Talip et al. (2016) [58]	Face to face focus groups	Thematic	Smoking only	Brunei 2017	0–4 years before
Tamvakas et al. (2010) [59]	Face to face semi-structured interviews with small groups (2/3 people)	Thematic	Smoking only	Greece 2010	0–4 years before
Tohid et al. (2011) [60]	Face to face focus groups and semi-structured interviews	Thematic	Smoking only	Malaysia 2019	10 + years before
Treacy et al. (2007) [61]	Face to face focus groups and semi-structured interviews	Thematic (inductive analysis)	Smoking only	Ireland 2004	5–9 years before
Turner et al. (2006) [62]	Face to face single sex focus groups	Thematic	Smoking only	UK (Scotland) 2006	0–4 years before
Vasquez et al. (2018) [63]	Face to face focus groups	Thematic	Smoking only	USA (Texas) No comprehensive ban	10 + years before
Woodgate et al. (2015) [64]	Face to face semi-structured interviews, participatory method ‘Photovoice’ and focus groups	Thematic	Smoking only	Canada (Western Canadian Province, unclear which) 2004, 2005, 2008, 2008	After
Yuksel et al. (2005) [65]	Face to face focus groups	Thematic and content analysis	Smoking only	Turkey 2009	10 + years before

**Fig. 2:**
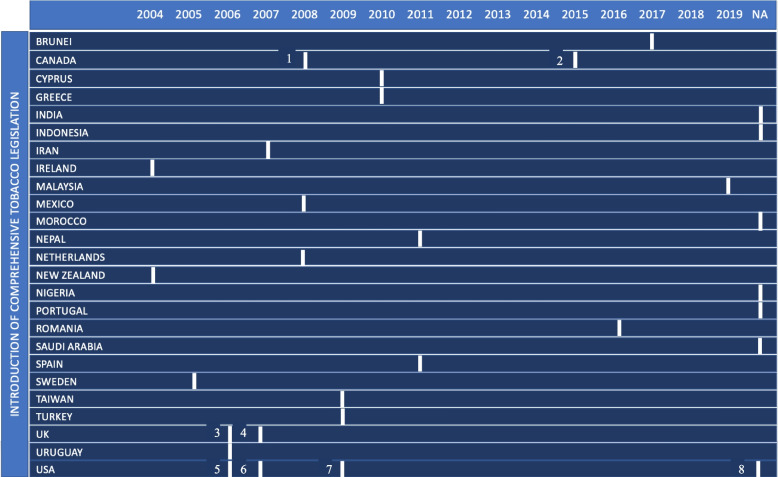
Year of introduction of comprehensive tobacco legislation by country/region. 1= British Columbia and Western Canadian Province, 2= Toronto and Vancouver, 3= Scotland, 4= England and Northern Ireland, 5= Hawaii, 6= Utah, 7= Newbraska, 8= Texas

Of the 41 studies, seven were based in the United Kingdom, four in the USA, four in Canada, two in India, three in Iran, two in the Netherlands, and one from each of the following countries; Uruguay Romania, Morocco, Portugal, Taiwan, Cyprus, Turkey, Ireland, Malaysia, Greece, Brunei, Sweden, Mexico, Nigeria, Spain, New Zealand, Nepal, Saudi Arabia, and Indonesia. For the purpose of this study, comprehensive tobacco legislation was defined as legislation banning smoking in all public spaces, including bars and restaurants and data were obtained from www.tobaccocontrollaws.org. This legislation was introduced within the 41 included studies between 2004 and 2019, with seven studies being conducted in countries, or regions within countries, that still have no comprehensive tobacco legislation in place. See Fig. 2 for the year of introduction of comprehensive tobacco legislation by country/region.

All studies focused on young people, with participants aged between 10 and 19 years. Thirty-two of the included studies employed focus groups, 19 face to face semi-structured interviews, one small group semi-structured interview, one telephone semi-structured interview, one unstructured face to face interview, one ethnography and one written narrative.

### Quality assessment

Seventeen included studies were rated as high, 19 medium and five low quality using the Evidence for Policy and Practice Information and Co-ordinating Centre (EPPI-centre) tool for the appraisal of qualitative studies [23]. The majority of high quality studies came from the following high income countries; the USA, UK, Canada, the Netherlands and Ireland, whilst only three were based in lower and middle income countries; India, Iran, and Morocco. Moreover, 14 out of 17 high quality studies, as well as all five low quality studies were conducted before the introduction of comprehensive tobacco legislation. The detailed quality assessments are available in Additional file 3.

### Exploration of stakeholder views on adolescent smoking

**Fig. 3:**
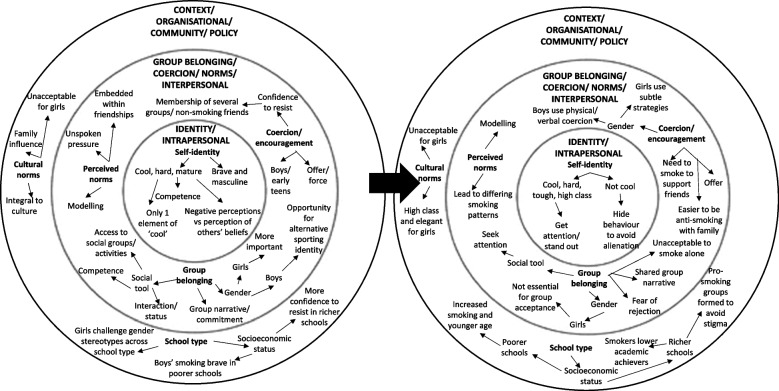
Themes mapped onto the social ecological model before and after the introduction of comprehensive smoking legislation

Synthesis resulted in the conceptualisation of five themes, which link to the review’s research questions and broadly map onto the socio ecological model [24]; context: culture and socioeconomic status, perceived norms and modelling, perceived control, coercion and encouragement, group belonging and social selection, and identity construction and performance (see Fig. 3), which are all perceived to interact to affect peer influence processes. The contributions of each study to the themes are detailed in Additional file 4.

#### Context: culture and socioeconomic status: before the introduction of comprehensive legislation

This theme focuses on the higher level determinants which set the wider context and interact with the lower level determinants discussed in the subsequent four themes to affect smoking behaviour. Nineteen studies published before the introduction of comprehensive tobacco legislation contributed to this theme [25, 26, 29, 30, 34, 36, 43–46, 48, 53, 54, 57, 59, 61–63, 65]. The main findings within this theme centred around culture and socioeconomic status.

The first key determinant was cultural norms. Family were generally seen to exert a stronger influence on adolescents who were from ethnic minorities [43, 59], compared to those from a white ethnic group. Moreover, it was perceived to be socially unacceptable for girls to smoke in some cultures. For example, one study [43] collected data in Morocco, finding that girls were more confident to resist smoking due to the unacceptability of girls’ smoking in society. In contrast, another study found that smoking was a desirable behaviour among adolescent males [45]. Adolescent male smokers in Saudi Arabia were perceived to be influenced by a need to look ‘Western’ and ‘civilised’, although there were contrasting opinions on whether smoking would help to achieve that [45].

In contrast, smoking was viewed as an integral part of the culture where adolescents were perceived to be surrounded by smoking. This perception of high smoking prevalence and cultural norms was perceived to have an important influence on whether an individual started smoking. For example, in Tamvakas [59], smoking was seen as an integral part of the Greek culture.

Results also touched upon findings according to school culture, with one study showing that girls smoking to portray a ‘hard’ image and compete with boys was consistent across school type from an inner city deprived school to a suburban predominantly middle class school [30].

Further results related to socioeconomic status. For example, students attending poorer government schools in Morocco perceived boys’ smoking to be brave, and students were exposed to a higher prevalence of smoking among parents. Whereas students attending richer private schools with higher quality teaching, lower smoking prevalence and lower exposure, were perceived to have more confidence to resist pressure [43]. Smoking was also perceived to be determined by the lack of structured activities available for adolescents within poorer areas [61], as well as taking part in weekend cultural leisure activities with friends that are associated with smoking, such as going to discos.

Moreover, school level differences between schools of a similarly low socioeconomic status were observed according to network structure and culture around smoking. A school with more friendship groups was perceived to have a higher level of smoking and a more favourable perception of smoking [62].

Overall, this suggests that the social processes by which adolescents take up smoking differ according to a mixture of school type, peer group structure, socioeconomic composition and the smoking culture within the school, as well as the wider cultural context.

#### Context: culture and socioeconomic status: after the introduction of comprehensive legislation

Six studies published after the introduction of comprehensive legislation contributed to data on contextual determinants [27, 39, 41, 49, 52, 55]. Again, contextual themes comprised of culture, identity and socioeconomic status. Smoking was frequently perceived to be linked to those of a lower socioeconomic status, with the age of initiation reported to be younger amongst groups of a lower SES and linked to poorer academic outcomes [49].

In terms of culture, in certain countries, such as Iran, there were contradictory perceptions of smoking for girls, such as ‘high class’ and ‘elegant’ versus stigmatised, immoral and unacceptable [27, 39]. Confidence to resist peer influence was varied and dependent upon context, such as the cultural acceptance of girls’ smoking [52].

School type related to socioeconomic status and smoking prevalence. Within communities and schools of a higher socioeconomic status and a very low smoking prevalence and normalisation, individuals were negatively evaluated for smoking. In turn, this affected the way smoking occurred in groups, with adolescents avoiding smoking at school due to feelings of shame and fear of negative evaluation [41] or creating pro-smoking groups to avoid stigma, resulting in magnified isolation and stigmatisation [55].

Overall, culture and socioeconomic status were perceived as important contextual determinants both before and after the introduction of comprehensive legislation. Data available after the introduction of comprehensive legislation, in a more denormalised tobacco smoking context, described changes in social interactions around smoking to cope with its stigmatisation, particularly relating to the perceived association between smoking and a lower socioeconomic status within affluent schools.

#### Perceived norms and modelling: before the introduction of comprehensive legislation

This theme relates to how individuals perceive the smoking related attitudes and behaviours of their peers, with 18 contributing studies published before the introduction of comprehensive tobacco legislation [26, 32, 34, 42–45, 47, 50, 53, 54, 56, 58–63]. The main findings within this theme showed that indirect influence also contributes to smoking behaviour among adolescents, through their perception of smoking norms.

Examples of indirect influence were confined to an unspoken pressure to smoke due to perceptions of smoking as the norm, with perceived high prevalence and positive attitudes towards smoking among friends. Adolescents reported that smoking is a habit embedded within friendships and linked to having friends who are smokers [44, 60]. They reported that access to cigarettes was easier and there was a will to smoke in order to not feel inferior to their smoking friends and to search for social identity [58].

Findings showed that older adolescents model smoking behaviour, and that adolescents feel confusion and tension when confronted with peer smoking and expectations, which often contrasts with family expectations of refraining from smoking [63]. Modelling was also reported to exert influence on adolescents’ decisions to smoke, with those with parents who smoke being more likely to smoke themselves [32, 34]. These adolescents also reported having easier access to cigarettes and perceiving smoking as a normal part of adulthood [32]. Conversely, one study found that those who had smoking parents were more likely to perceive this as a reason to avoid smoking, and to avoid modelling smoking to younger children [53]. Other influences were teachers who, in one study, were perceived to tolerate smoking among adolescents, as long as it took place away from school buildings [47].

The influence of male family members, such as fathers and older brothers, on boys’ smoking behaviour was deemed to be particularly important in Saudi Arabia [45].

Overall, adolescents’ perceptions of peer smoking norms, as well as behaviour modelled by parents and older adolescents were important determinants of smoking behaviour. These factors align with the contextual findings discussed above, which demonstrated that cultural and socioeconomic determinants influenced the extent to which smoking was perceived as the norm in different contexts. This may influence the extent to which modelling may affect smoking behaviour.

#### Perceived norms and modelling: after the introduction of comprehensive legislation

Four studies published after the introduction of comprehensive legislation reported perceived norms as being key to smoking behaviour [39, 49, 52, 55].

As with studies published before the introduction of comprehensive legislation, perceived norms were perceived to indirectly influence smoking behaviour [39, 49, 52, 55]. However, perceived norms were also thought to impact upon adolescent smoking patterns. For example, when school-level prevalence was low, this didn’t necessarily encourage the uptake of smoking, but it did pressurise those who smoke to operate outside of the school cohort’s mainstream culture, with smokers seeking a low profile or attending smoking friendly social events. Whereas, in a high smoking context, smoking took place in the school, with little fear of judgement by peers [55].

Modelling by parents and older siblings, as well as older peers, was also seen to contribute to perceptions of norms and subsequent smoking [49, 52]. Gender differences were also identified, with girls perceived to be more likely to emulate smoking behaviour of individuals who are important to them, whereas boys were perceived to emulate older individuals [49].

Overall, after the introduction of comprehensive legislation, smoking was viewed as a less normative behaviour. Thus, the perceived norms of the school were reported to impact upon where smoking took place and the extent to which adolescents made an effort to do this covertly to avoid negative judgement. This finding relating to perceived norms aligns with the findings within the context: culture and socioeconomic status theme, which demonstrated that this negative judgement varied according to cultural and socioeconomic norms across different countries and school settings.

#### Perceived control, coercion, and encouragement: before the introduction of comprehensive legislation

This theme relates to the interpersonal determinants of smoking behaviour in relation to control, coercion, and encouragement from peers, with 29 contributing studies published before the introduction of comprehensive tobacco legislation [25, 26, 28–36, 40, 42–48, 50, 54, 56–58, 60–63, 65]. The main findings within this theme showed that, intertwined with the need to belong to a group, was direct peer influence.

Direct peer influence, manifested through control, coercion, and encouragement was reported by the majority of studies [25, 26, 28–36, 40, 42–48, 50, 54, 56–58, 60–63, 65]. Most descriptions involved acts, such as being offered cigarettes or even forced, with an unspoken pressure to accept or be subject to social exclusion or ridicule [56]. This evidence of direct peer influence was contradicted by a belief that adolescents can say no to this pressure without any repercussions, if surrounded by real friends [30]. Pressure was perceived to be more prevalent among early teens and males, who were reported to be directly pressured to smoke to conform with a masculine identity [65]. Moreover, there were reports of individuals being ridiculed for refusing to accept a cigarette and a perception of a lack of refusal skills among adolescents [34, 45].

There were also reports from one study that older students may derive status from directly influencing younger students to emulate their smoking behaviour [36]. Several studies found that the need to fit in was competing with the need to also stand out as an individual. Moreover, belonging to a non-smoking peer group was shown to facilitate adolescents’ confidence to resist coercion to smoke [29] and an individual’s membership of several different peer groups diluted peer influence [31]. Membership of several peer groups reduced the need to smoke to achieve group belonging.

Overall, direct peer influence was a prevalent theme amongst studies. This was manifested in different ways, as a coercive process. Protective factors included belonging to multiple peer groups or to one non-smoking peer group.

#### Perceived control, coercion, and encouragement: after the introduction of comprehensive legislation

Eight studies published after the introduction of comprehensive legislation reported smoking as being key to group belonging and social selection [27, 37, 39, 41, 49, 52, 55, 64]. As with studies published before the introduction of comprehensive legislation, pressure was consistently reported from peers by many studies, particularly in social settings.

For some, being offered cigarettes in a group setting was seen to exert pressure on individuals to conform [49, 52], whilst others reported subtle forms of influence and even feeling the need to support their smoking friends [64].

Pressure to smoke was perceived to manifest differently according to gender, with boys being more likely to be physically or verbally coerced, and girls more likely to adopt subtle strategies to encourage their peers to smoke [49].

Individuals were reported to differ in their ability to resist peer pressure in terms of the confidence expressed and it was reported to be easier to express anti-smoking sentiment to parents and family, rather than peers [52].

Overall, social influence in the form of control, coercion and encouragement was important in both a pre- and post- legislative context. After the introduction of comprehensive legislation, girls were reported to use more subtle coercion techniques. According to the previous themes, gender norms varied according to culture, thus these themes may interact to affect the manner in and extent to which different genders are influenced by their peers.

#### Group belonging and social selection: before the introduction of comprehensive legislation

Thirty studies reported smoking as being key to group belonging and social selection [25, 28–33, 35, 36, 38, 40, 42–48, 50, 51, 53, 54, 56, 57, 59–63, 65]. This theme relates to the interpersonal determinants of smoking behaviour in relation to the need to be accepted and belong to a group and social selection, whereby individuals choose their group of friends according similarity in smoking status.

Within twenty-five studies, smoking was seen as a way to facilitate increasing popularity, creating a social identity and gaining acceptance into a group through the creation of shared activities and experiences [25, 28–32, 35, 36, 38, 40, 44–48, 50, 53, 54, 56, 57, 59, 60, 62, 63, 65]. Specifically, smoking was perceived to allow individuals to mix with older children, as well as accessing a wider variety of social groups [53, 57, 59]. This suggests that smoking may be used by adolescents as a tool to facilitate social interaction and status, as opposed to being an inherently enjoyable activity. Indeed, within many of the included studies, smoking was perceived consciously as a social tool allowing adolescents to converse, connect and feel less awkward in a social setting [40, 59]. Some adolescents even described forcing themselves to acquire the taste so that they were able to make use of this social tool [40].

Others showed adolescents to have a sophisticated understanding of smoking as a tool to avoid rejection and create a shared narrative among group members as well as other factors such as showing commitment to the group and developing outgroup discrimination for those who do not smoke [28]. Reports of the use of smoking as a social tool are linked to social selection, or adolescents choosing friends according to their smoking status, with reports of adolescents who wish to smoke, subsequently seeking out smoker friends [30]. Smoking was also used as a tool was to gain entrance to new social groups and start new conversations and to participate in cultural activities outside of school, such as clubbing [38]. Thus, the use of smoking as a tool to facilitate group belonging, is likely to vary according to context. However, as highlighted in the section above, smoking was only perceived to facilitate social acceptance when the individual was a competent and confident smoker, otherwise the act could have the opposite effect of undermining their group acceptance [36, 42].

Group belonging and identity, alongside the process through which smoking was integrated into friendships, were found to be more important for girls, where smoking and sharing cigarettes allowed them to fully engage in group activities, create a group identity, and create a balance between obtaining social capital and being stigmatised for smoking [25, 56]. For example, girls reported smoking being linked to social cohesion and trust to reinforce social bonds, bound by willingness to share cigarettes, whereas boys were more likely to go to extreme measures to get money for their own cigarettes and were averse to sharing. Moreover, boys reported smoking to portray an image consistent with group members, but also reported having the opportunity for avoiding smoking through the creation of alternative identities around activities, such as sport. Whereas girls were more likely to spend break times undertaking sedentary activities [51]. Further to this, girls were also more likely to associate, be romantically involved with and be influenced by older boys and to have to accept a lower status if they decided not to smoke [48].

Overall, prior to the introduction of comprehensive legislation, where smoking was more normalised, smoking behaviour was viewed as an important tool to enhance adolescents’ group belonging and popularity. Again, relating back to the findings reported within the previous themes, the use and effectiveness of smoking as a social tool may vary according to cultural norms, such as the social acceptability of girls’ smoking.

#### Group belonging and social selection: after the introduction of a comprehensive smoking ban

Seven studies published after the introduction of comprehensive legislation reported smoking as being key to group belonging and social selection [27, 37, 39, 41, 49, 55, 64]. The main findings within this theme, like the findings from before the introduction of comprehensive legislation, demonstrated that adolescents perceived smoking to be key to group acceptance, while refusing to smoke could result in rejection from a group. Thus, adolescents reported being afraid to say no, or not to conform, due to the perceived risk of losing friendships and the associated support network [37, 41, 55].

This was reflected in adolescents reporting the need to smoke in order to belong to a group [41, 49]. It was viewed as awkward to smoke alone, for example, adolescents would wait for school breaks when a group could congregate [55]. Students reported getting into a routine of smoking with friends, which would then lead to making good memories and a group atmosphere. This was perceived to reinforce smoking behaviour, despite awareness of the health risks [55].

In contrast, other findings showed that girls felt smoking was not essential for group membership [55] and that individuals valued health over and above the need to belong to a group, and that non-smokers deselected smoker friends [64]. A further study found more boys to report smoking in groups than girls [49]. There was also evidence from only one study, based in Iran, to suggest that smoking was used as a tool to achieve adolescents’ social needs [27].

To summarise, before comprehensive legislation was introduced, and smoking was more normalised, smoking was strongly perceived to be key to group acceptance and popularity. Whereas, after the introduction of comprehensive legislation, where smoking was more denormalised, smoking was not always a prerequisite for group membership, reports of the use of smoking as a social tool were less prevalent and smoking behaviour was not always strongly perceived to be linked to group acceptance and popularity. This decreased prevalence aligns with the findings discussed within the context: culture and socioeconomic status theme, which demonstrated that after the introduction of comprehensive tobacco legislation social acceptability of smoking varied according to school-level socioeconomic status. Thus, the social selection and group belonging processes described above would vary according to contextual determinants.

#### Identity construction and performance: before the introduction of comprehensive legislation

Twenty three studies reported smoking as contributing to identity construction and performance [30, 32–36, 38, 40, 42, 43, 45, 46, 48, 50, 51, 53, 54, 56, 57, 61–63, 65]. Identity construction was seen as the perception of the role of smoking in facilitating the formation of a certain identity. Whilst performance relates to the act of using smoking related symbolism, such as the act of smoking, appearing to smoke or carrying cigarettes. These identities and the associated behaviour can both be influenced by others or initiated by individuals who then select friends with similar identities [66].

The majority of studies focused on smoking as a way of creating a self-identity at an important stage of development. Mainly, this was manifested in adolescents reporting smoking to look cool, hard [30, 32, 33, 35, 38, 40, 42, 50, 51, 56, 61–63], mature [43, 45, 46, 48, 62, 63, 65] or popular [38, 51, 57, 62, 63]. With males in particular aiming to portray a brave and masculine identity [33, 34, 45, 53, 65].

However, opinions differed on whether smoking was actually perceived as an activity undertaken by popular or ‘cool’ individuals or not. For example, individuals reported negative personal perceptions of smoking [62], whilst reporting a belief that others perceive cigarettes as cool, good for them and fun [63]. Thus, this misperception may work to perpetuate the perceived need to smoke to look cool. Indeed, the perception of smoking as cool was seen by some to be more important in influencing smoking behaviour than peer influence. It was reported that smoking could carry both a high and a low status as it was just one element of being cool, rather than a measure of ‘cool’ in itself [51].

Other factors, such as ethnicity and gender were also reported to affect smoking behaviour. For example, girls were motivated by trying to look mature and by using smoking as a tool to overcome traditional female stereotypes and assert equality by competing with boys [38].

One study highlighted that smoking awkwardly or symbolic smoking through techniques such as pretending to inhale could actually do more harm than good to an individual’s social status [36]. Others reported that smoking was simply an activity that they engage in, not something that was perceived as key to identity [54].

Overall, the majority of studies found smoking and its associated performative acts to be key to adolescent identity construction. Opinions differed on the extent to which smoking was perceived as ‘cool’, but the majority perceived this to be the case [30, 32, 33, 35, 38, 40, 42, 50, 51, 56, 61–63].

#### Identity construction and performance: after the introduction of comprehensive legislation

Six studies published after the introduction of comprehensive legislation reported smoking as being part of identity construction and performance [27, 39, 41, 49, 55, 64]. The main findings within this theme showed that a number of individual determinants contributed to adolescents’ decision to start, and continue, to smoke, with a large proportion of the data focusing on smoking as a way of developing a sense of identity. Much like the findings from before the introduction of comprehensive legislation, although somewhat less prevalent, reasons cited included trying to appear ‘cool’ [41, 64]. Appearing ‘cool’ was found to be a key motivatior where adolescents attended a school with a high smoking prevalence, with one study citing girls and boys smoking to appear ‘hard’ or ‘tough’ or ‘high class’ [41].

Others suggested that smoking was not perceived as cool, particularly in a society where smoking has become denormalised and the adverse health effects are so well known. Smoking was instead overwhelmingly perceived as something which caused adolescents to be alienated from school culture [64]. It was also perceived as a behaviour deserving of sympathy due to signalling unhappiness in an adolescent’s life [64]. This sentiment was echoed in other studies where adolescent smokers discussed the need to hide their smoking from peers for fear of being judged negatively [55].

Others suggested smoking was a way to get attention and stand out from the crowd and can often be used as a largely symbolic activity by carrying cigarettes, without fully engaging in the activity. This symbolism varied according to countries, with data from Iran finding that participants perceived smoking to be a symbol of being high class or sophisticated [27, 39].

Overall, the data from after the introduction of comprehensive smoking legislation, in a more denormalised context, reports more negative perceptions of smoking and outlines the social risks, such as negative judgement from peers, of engaging in the behaviour. Whilst the data from before the introduction of comprehensive legislation found some individuals to perceive smoking negatively, the data did not reflect this as a wider opinion. These findings align with the findings described within the above themes. For example, the contextual determinants, as well as lower perception of smoking as the norm in a more denormalised tobacco smoking context would combine with identity construction to determine a lower likelihood of the use of smoking to portray a ‘cool’ image and of individuals being influenced to smoke in order to be perceived as ‘cool’.

## Discussion

This meta-ethnography is the first study to demonstrate, drawing on international data, that peer processes relating to adolescent smoking may undergo changes as norms for smoking within society change. Overall, findings showed that adolescents’ fears of negative judgement due to smoking were more commonly reported in a more denormalised tobacco smoking context. Whilst adolescents also less commonly reported using smoking as a social tool to facilitate group belonging, social status and gender equality within a more denormalised tobacco smoking context.

Social influence and selection were reported to occur across tobacco smoking normalisation contexts, both before and after the introduction of comprehensive smoking legislation. However, the social groupings in which control, coercion and encouragement occurred differed within normalised and denormalised contexts, occurring in the mainstream school culture within normalised contexts, but mainly occurring within groups alienated from the mainstream culture within denormalised contexts. This continued importance across temporal contexts, suggests that both processes should be considered within future intervention development, but that this should be adapted according to the level of tobacco denormalisation. Currently, interventions tend to focus on education as well as harnessing social influence in a positive manner to facilitate adolescents to exert influence on peers not to take up smoking, or to quit if they have already taken up the habit [7].

Gender, cultural determinants and school-level socioeconomic context were reported to be important across tobacco smoking normalisation contexts. Despite this, results relating to socioeconomic status were sparse. Only 17 out of 38 studies reported students’ SES, six studies focused on participants mainly from deprived communities [26, 41, 42, 61–63] and only four studies assessed results separately according to school-level SES [33, 43, 52, 55].

Results of the synthesis conducted in a more normalised tobacco smoking context consistently showed evidence of adolescents using cigarettes as a social tool. Reports of using cigarettes as a social tool differed after the introduction of comprehensive legislation, in a more denormalised tobacco smoking context. These differences included increased discussion of how smoking was not an essential factor for group membership and only one study reporting the use of smoking as a social tool. These results could be explained by the fact that smoking is reported to become increasingly stigmatised within societies where smoking has become denormalised. Thus, aligned with the findings of the current review, regular smoking instead becomes a socially unacceptable behaviour which tends to occur within groups of smokers, and covertly to avoid the attached stigma [67, 68]. Thus, these contextual issues may contribute to the perpetuation of socioeconomic inequalities in smoking and marginalisation as a result of smoking [41].

Current interventions do not account for the differing processes occurring within different school contexts reported within this review. These include differing socioeconomic composition, culture, social norms relating to smoking and subsequently differing smoking behaviour, such as whether smoking takes place as a central or peripheral activity. These interventions may therefore miss opportunities to effectively target those of a lower socioeconomic status, both at a school level and an individual level, such as individuals from a lower SES attending affluent schools [10]. This is consistent with a previous systematic review which found that only one in four health behaviour interventions mentioned SES inequalities. A recommendation was made for the need for routine testing of the effects of future interventions on inequalities [69]. Both the mechanisms of identifying which pupils to train as peer supporters (i.e. who will exert social influence), and training provided to peer supporters about interacting with other students (i.e. how peer supporters are selected into social groups) could differ according to school context. Further research is required to focus upon differences between school contexts and how we can adapt interventions to enhance their effectiveness within different schools in contexts where smoking has now become denormalised [70]. For example, A Stop Smoking in Schools Trial (ASSIST) Global states that the intervention is likely to work within low income countries where smoking remains normalised [71].

Results for the synthesis focused on more normalised tobacco smoking contexts showed reports of girls using cigarettes as a tool to strive for gender equality, through strategies such as trying to portray a ‘hard’ image [38]. Reporting of this did not differ according to SES. One explanation for this could be that smoking was still normalised within society and, thus, smoking as cool still dominated across SES settings. This was not reported within studies conducted after the introduction of comprehensive legislation, within more denormalised tobacco smoking contexts.

Parental modelling was reported to be an important influence on smoking among adolescents in both normalised and denormalised tobacco smoking contexts. This is consistent with Previous studies which have shown adolescents from a lower SES to experience increased exposure to parental smoking in comparison with their affluent peers [9]. Thus, this may contribute to the perpetuation of inequalities in a context where overall levels of smoking are reducing, but more slowly among lower SES groups [12].

The results of this study are aligned with the sister review of quantitative social network effects on adolescent smoking. With a focus on network characteristics, findings showed variation in the composition and effect of network characteristics on smoking across different types of school, including those differing according to socioeconomic status and other characteristics [11]. Conclusions were aligned with the current review, revealing the lack of focus on socioeconomic status and the need for future research to employ these methods to understand how network structure and its influence on adolescent smoking may differ across school types.

### Strengths and limitations

The main strengths of this systematic review are the thorough review processes undertaken, such as double screening and quality assessment. This review only identified eight eligible studies [27, 37, 39, 41, 49, 52, 55, 64] that were conducted after, compared to 31 studies [25, 26, 28–36, 38, 40, 42–48, 50, 51, 53, 54, 56–63, 65] conducted before the introduction of comprehensive smoking legislation. All eight of these studies were conducted between two and ten years post-legislation. Researchers who conceptualise schools as complex systems have consistently advocated for longer follow-up periods of at least ten years within studies to allow any changes to become embedded within the system [72]. Thus, a larger volume of future research is required to focus on social influence processes within contexts at least ten years after the introduction of such legislation. This would help to obtain a greater insight into how the denormalisation of tobacco smoking has altered social influence processes within school systems. In addition, the use of a proxy measure to understand denormalisation may have affected the accuracy of the results, through restricting the ability to understand different levels of denormalisation, as opposed to treating normalisation and denormalisation as dichotomies. A more specific measure would have allowed for differentiation between levels of denormalisation, although this was beyond the scope of the current review.

Further to this, there are several reasons why results should be interpreted with caution. The heterogeneity of study characteristics, including methods, sample size and characteristics and culture, make direct comparisons between studies difficult. There was also a lack of diversity between studies, with the majority of evidence coming from high income countries. While information on e-cigarette use was beyond the scope of the study, this is an important contextual issue for cigarette smoking that should be considered within future studies and systematic reviews.

#### Conclusion

Within the context of tobacco smoking denormalisation, fears of negative judgement and stigma related to smoking have increased among adolescents, and the use of smoking as a social tool has decreased. Both social influence and selection and school level SES have maintained their importance in perceived differentiated processes across contexts. A greater volume of future research should ensure a measurement and focus on SES both at the individual and school level, gender and cultural contexts, and focus on contexts where comprehensive legislation has been introduced for at least ten years, thus further accelerating denormalisation. This would facilitate an enhanced understanding of how differences across school-level SES contexts manifest once post-legislative norms have been established. Subsequently, this would allow future interventions to be adapted to different school contexts to tackle inequalities.

## Supplementary Information

**Additional file 1.** Medline search strategy.

**Additional file 2.** Synthesis categories.

**Additional file 3.** Contribution of each study to themes.

**Additional file 4.** Final quality assessments.

## Data Availability

Quality assessments and study contributions to themes are available in the Appendices.

## Abbreviations

ASSIST: A Stop Smoking In Schools Trial
ASSIA: Applied Social Sciences Index and Abstracts
BEI: British Education Index
CINAHL: Cumulative Index to Nursing and Allied Health Literature
EPPI-centre: Evidence for Policy and Practice Information and Co-ordinating Centre
ERIC: Education Resources Information Center
ETHOS: E-Theses Online Service
NA: Not Applicable
PRISMA: Preferred Reporting Items for Systematic reviews and MetaAnalyses
PROSPERO: Prospective Register of Systematic Reviews
SABM: Stochastic ActorBased Models
SES: Socioeconomic Status
SNA: Social Network Analysis
SPIDER: Sample, Phenomenon of Interest, Design, Evaluation, Research type
SURE: Specialist Unit for Review Evidence
