# Supplementary material for: Perceptions of friendship, peers and influence on adolescent smoking according to tobacco control context: a systematic review and meta-ethnography of qualitative research
Source: BMC Public Health. 2023 Mar 3;23:424. doi: 10.1186/s12889-022-14727-z (PMC9983235; doi:10.1186/s12889-022-14727-z)
Supplement: Supplementary file 1 — Additional file 1. Medline search strategy. [file 12889_2022_14727_MOESM1_ESM.docx]

Appendix 1: Medline search strategy

1 smoking/

2 exp cigarette smoking/

3 smoking.ti,ab.

4 ((smok* or tobacco or cigarette* or nicotine) adj3 (addict* or use* or usage or using or intake or consum*)).ti,ab.

5 ((smok* or tobacco or cigarette* or nicotine) adj3 (prevalence or behavio?r)).ti,ab.

6 or/1-5

7 exp Adolescent/ or exp Minors/ or exp Young Adult/

8 (student* or pupil* or young adult* or young person or juvenile* or young people ).tw.

9 (adolesc* or teen* or minor* or boy* or girl* or youth*).tw.

10 or/7-9

11 Peers/ or peer relations/ or peer influence/ or peer group/ or friends/ or friendship/ or interpersonal relations/

12 social behaviour/ or social capital/ or social isolation/ or social norms/ or social groups/

13 social behavio?r.tw

14 ((peer* or friend*) adj3 (pressure or influence or selection or norm* or role)).mp.

15 or/11-14

16 exp Qualitative research/

17 qualitative research.mp.

18 Interview/ or interview.mp.

19 ((interview* or survey or focus group*) adj3 qualitative).mp.

20 focus groups/ or focus group.mp.

21 (qualitative adj3 (study or research or method* or analysis or cod* or them* or question*1 or

data)).ti,ab.

22 (thematic analysis or ethnological research or ethnograph*).ti,ab.

23 (theme*1 adj2 (qualitative or analysis or coding or codes or grouping or identif*)).ti,ab.

24 (grounded adj (theor* or study or studies or research or analys?s)).mp.

25 (data adj1 saturat*).ti,ab.

26 (field adj (study or studies or research)).ti,ab.

27 (perception* or thought* or knowledge or perspective* or attitude* or opinion* or idea* or

"point of view" or belie* or viewpoint* or understand* or representation* or views or

experience* or behaviour or behaviour or comprehend or comprehension or know*or

aware*).mp.

28 or/16-27

29 6 and 10 and 15 and 28

30 limit 29 to yr="1997-current
