# Supplementary material for: Perceptions of friendship, peers and influence on adolescent smoking according to tobacco control context: a systematic review and meta-ethnography of qualitative research
Source: BMC Public Health. 2023 Mar 3;23:424. doi: 10.1186/s12889-022-14727-z (PMC9983235; doi:10.1186/s12889-022-14727-z)
Supplement: Supplementary file 2 — Additional file 2. Synthesis categories. [file 12889_2022_14727_MOESM2_ESM.docx]

Appendix 2 : Synthesis categories

| Synthesis category | | Included studies (author and year) |
| --- | --- | --- |
| 10+ years before/no smoking ban introduced | High quality | El Kazdouh (2018)  Johnson (2003)  Mishra (2005)  Vasquez (2018) |
|  | Low-medium quality | Arora (2010)  Craciun (2008)  Fithria (2021)  Mutaz (2020)  Nwafor (2005)  Sanchez Martinez (2008)  Stjerna (2004)  Tohid (2011)  Yuksel (2005) |
| 5-9 years before smoking ban introduced | High quality | Baillie (2005)  Denscombe (2001a)  Denscombe (2001b)  Milton (2008)  Plano Clark (2002)  Stewart Knox (2005)  Treacy (2007) |
|  | Low-medium quality | Dijk (2006)  Ioannou (2010)  Plumridge (2002) |
| 0-4 years before smoking ban introduced | High quality | Amos (2007)  Niknami (2008)  Turner (2006) |
|  | Low-medium quality | Fraga (2011)  Haines (2009)  Mitschke (2008)  Perez Milena (2012)  Rothwell (2011)  Talip (2016)  Tamvakas (2010) |
| After smoking ban introduced | High quality | Lewis (2003)  Schreuders (2019)  Woodgate (2015) |
|  | Low-medium quality | Baheiraei (2017)  Hong (2015)  Jafari (2022)  Peterson (2019)  Povlsen (2018) |
