# Supplementary material for: Perceptions of friendship, peers and influence on adolescent smoking according to tobacco control context: a systematic review and meta-ethnography of qualitative research
Source: BMC Public Health. 2023 Mar 3;23:424. doi: 10.1186/s12889-022-14727-z (PMC9983235; doi:10.1186/s12889-022-14727-z)
Supplement: Supplementary file 4 — Additional file 4. Final quality assessments. [file 12889_2022_14727_MOESM4_ESM.docx]

Table 1. Before the introduction of comprehensive tobacco legislation: Contribution of each study to themes

|  | Context: culture and socioeconomic status: Before the introduction of comprehensive legislation | Perceived norms and modelling: Before the introduction of comprehensive legislation | Perceived control, coercion, and encouragement: Before the introduction of comprehensive legislation | Group belonging and social selection: Before the introduction of comprehensive legislation | Identity construction and performance: Before the introduction of comprehensive legislation |
| --- | --- | --- | --- | --- | --- |
| Amos et al. (2006) |  |  |  |  |  |
| Arora et al. (2010) |  |  |  |  |  |
| Baillie et al. (2005) |  |  |  |  |  |
| Craciun et al. (2008) |  |  |  |  |  |
| Denscombe et al. (2001) |  |  |  |  |  |
| Denscombe et al. (2001b) |  |  |  |  |  |
| Dijk et al. (2006) |  |  |  |  |  |
| El Kazdouh et al. (2018) |  |  |  |  |  |
| Fithria et al. (2022) |  |  |  |  |  |
| Fraga et al. (2011) |  |  |  |  |  |
| Haines et al. (2009) |  |  |  |  |  |
| Ioannou et al. (2010) |  |  |  |  |  |
| Johnson et al. (2003) |  |  |  |  |  |
| Milton et al. (2008) |  |  |  |  |  |
| Mishra et al. (2005) |  |  |  |  |  |
| Mitschke et al. (2008) |  |  |  |  |  |
| Mutaz et al. (2020) |  |  |  |  |  |
| Niknami et al. (2008) |  |  |  |  |  |
| Nwafor et al. (2012) |  |  |  |  |  |
| Perez-Milena et al. (2012) |  |  |  |  |  |
| Plano Clark et al. (2002) |  |  |  |  |  |
| Plumridge et al. (2002) |  |  |  |  |  |
| Rothwell et al. (2011) |  |  |  |  |  |
| Sanchez Martinez et al. (2008) |  |  |  |  |  |
| Stewart-Knox et al. (2005) |  |  |  |  |  |
| Stjerna et al. (2004) |  |  |  |  |  |
| Talip et al. (2016) |  |  |  |  |  |
| Tamvakas et al. (2010) |  |  |  |  |  |
| Tohid et al. (2011) |  |  |  |  |  |
| Treacy et al. (2007) |  |  |  |  |  |
| Turner et al. (2006) |  |  |  |  |  |
| Vazquez et al. (2018) |  |  |  |  |  |
| Yuksel et al. (2005) |  |  |  |  |  |

Table 2. After the introduction of comprehensive tobacco legislation: Contribution of each study to themes

|  | Context: culture and socioeconomic status: After the introduction of comprehensive legislation | Perceived norms and modelling: After the introduction of comprehensive legislation | Perceived control, coercion, and encouragement: After the introduction of comprehensive legislation | Group belonging and social selection: After the introduction of a comprehensive smoking ban | Identity construction and performance: After the introduction of comprehensive legislation |
| --- | --- | --- | --- | --- | --- |
| Baheiraei et al. (2017) |  |  |  |  |  |
| Hong et al. (2015) |  |  |  |  |  |
| Jafari et al. (2022) |  |  |  |  |  |
| Lewis et al. (2003) |  |  |  |  |  |
| Peterson et al. (2019) |  |  |  |  |  |
| Povlsen et al. (2018) |  |  |  |  |  |
| Schreuders et al. (2019) |  |  |  |  |  |
| Woodgate et al. (2015) |  |  |  |  |  |
| **TOTAL:** | 6 | 4 | 8 | 7 | 6 |
